# Nitric Oxide Is Required for L-Type Ca^2+^ Channel-Dependent Long-Term Potentiation in the Hippocampus

**DOI:** 10.3389/fnsyn.2016.00017

**Published:** 2016-06-29

**Authors:** Beatrice M. Pigott, John Garthwaite

**Affiliations:** The Wolfson Institute for Biomedical Research, University College LondonLondon, UK

**Keywords:** LTP, synaptic plasticity, hippocampus, L-type Ca^2+^ channel, NMDA receptor, nitric oxide, cGMP

## Abstract

Nitric oxide (NO) has long been implicated in the generation of long-term potentiation (LTP) and other types of synaptic plasticity, a role for which the intimate coupling between NMDA receptors (NMDARs) and the neuronal isoform of NO synthase (nNOS) is likely to be instrumental in many instances. While several types of synaptic plasticity depend on NMDARs, others do not, an example of which is LTP triggered by opening of L-type voltage-gated Ca^2+^ channels (L-VGCCs) in postsynaptic neurons. In CA3-CA1 synapses in the hippocampus, NMDAR-dependent LTP (LTP_NMDAR_) appears to be primarily expressed postsynaptically whereas L-VGCC-dependent LTP (LTP_L−VGCC_), which often coexists with LTP_NMDAR_, appears mainly to reflect enhanced presynaptic transmitter release. Since NO is an excellent candidate as a retrograde messenger mediating post-to-presynaptic signaling, we sought to determine if NO functions in LTP_L−VGCC_ in mouse CA3-CA1 synapses. When elicited by a burst type of stimulation with NMDARs and the associated NO release blocked, LTP_L−VGCC_ was curtailed by inhibition of NO synthase or of the NO-receptor guanylyl cyclase to the same extent as occurred with inhibition of L-VGCCs. Unlike LTP_NMDAR_ at these synapses, LTP_L−VGCC_ was unaffected in mice lacking endothelial NO synthase, implying that the major source of the NO is neuronal. Transient delivery of exogenous NO paired with tetanic synaptic stimulation under conditions of NMDAR blockade resulted in a long-lasting potentiation that was sensitive to inhibition of NO-receptor guanylyl cyclase but was unaffected by inhibition of L-VGCCs. The results indicate that NO, acting through its second messenger cGMP, plays an unexpectedly important role in L-VGCC-dependent, NMDAR-independent LTP, possibly as a retrograde messenger generated in response to opening of postsynaptic L-VGCCs and/or as a signal acting postsynaptically, perhaps to facilitate changes in gene expression.

## Introduction

Long-term potentiation (LTP) of CA3-CA1 glutamatergic transmission in the hippocampus is a widely studied example of synaptic plasticity considered to underlie certain aspects of learning and memory formation. The expression of LTP at these synapses can comprise multiple temporally and mechanistically distinct components that are located pre- and/or postsynaptically and found in relative isolation or in various combinations depending on the induction protocol and the period of observation (Raymond, 2007; Blundon and Zakharenko, 2008; Granger and Nicoll, 2014; Park et al., 2014). The clearest distinction is between LTP that is initiated by influx of Ca^2+^ through NMDA receptor (NMDAR) channels (Park et al., 2014) and LTP that can be elicited when NMDARs are blocked and which relies on the activation of L-type voltage-gated Ca^2+^ channels (L-VGCCs) in the postsynaptic neuron (Grover and Teyler, 1990). “Pure” NMDAR-dependent LTP (LTP_NMDAR_) generally displays the lowest induction threshold, develops within a few minutes and at least to begin with, involves post-translational alterations to postsynaptic proteins, including AMPA receptors. Under NMDAR blockade, eliciting L-VGCC-dependent LTP (LTP_L−VGCC_) needs patterns of stimuli that are relatively strong in terms of amplitude and/or frequency and/or duration, presumably reflecting the strong depolarization needed to activate the channels. It also has a relatively slow onset (peaking generally 10–30 min post-tetanus) and appears to be largely maintained by presynaptic changes. Without NMDAR blockade, many types of stimulation commonly delivered to afferent fibers can elicit a composite LTP in which LTP_NMDAR_ and LTP_L−VGCC_ coexist in varying proportions (Cavus and Teyler, 1996; Morgan and Teyler, 2001; Bayazitov et al., 2007; Grover et al., 2009), separable as early postsynaptic and late-onset presynaptic components (Zakharenko et al., 2001; Bayazitov et al., 2007). Moreover, by virtue of their depolarizing effect, activated NMDARs can contribute importantly to LTP_L−VGCC_ so that, without specific tests, the resulting LTP can masquerade as being LTP_NMDAR_ despite, in reality, being mixed (Blundon and Zakharenko, 2008; Padamsey and Emptage, 2014).

Nitric oxide (NO) participates in many forms of synaptic plasticity from early development into adulthood (Garthwaite, 2008; Steinert et al., 2010). Frequently integral to this role is the close functional link between NMDARs and NO generation, a link facilitated by the tethering of the neuronal isoform of NO synthase (nNOS) close to NMDARs such that the influx of Ca^2+^ associated with the opening of NMDAR channels, via calmodulin, stimulates the enzyme to synthesize NO from L-arginine (Garthwaite et al., 1988; Brenman et al., 1996). In hippocampal CA3-CA1 synapses, some early studies found that inhibition of NO synthesis reduced or blocked LTP from its earliest stages (Bohme et al., 1991; O’Dell et al., 1991; Schuman and Madison, 1991) but NO has more commonly been observed to participate in later phases of LTP, so that NO synthase inhibition results in a broadly unchanged early potentiation that gradually decays back towards baseline (Chetkovich et al., 1993; Boulton et al., 1995; Haley et al., 1996; Wilson et al., 1997; Kleppisch et al., 1999; Lu et al., 1999; Bon and Garthwaite, 2003; Hopper and Garthwaite, 2006; Phillips et al., 2008; Johnstone and Raymond, 2011). Whilst having postsynaptic actions relevant to hippocampal LTP (Lu et al., 1999; Serulle et al., 2007), its ability to diffuse isotropically from its site of formation also makes NO an attractive candidate as a retrograde trans-synaptic messenger that informs presynaptic nerve terminals when postsynaptic NMDARs are active (Garthwaite, 2016). Evidence from several brain areas is consistent with NO performing such a retrograde signaling role, leading to enduring alterations in neurotransmitter release (Hardingham et al., 2013).

In accordance with NO participating in later phases of LTP, and with it performing a retrograde messenger role, a single burst of stimuli delivered to CA3-CA1 synapses at the theta rhythm (5 Hz) generated an apparently pure LTP_NMDAR_ that decayed almost back to baseline over 160 min and was not associated with presynaptic changes, as determined by the rate of loss of the fluorescent dye FM 1–43 from CA3 nerve terminals, nor was it affected by inhibition of NO synthase (Johnstone and Raymond, 2011). Increasing the number of trains of theta-burst stimuli led to an increasingly persistent LTP whose maintenance depended on NO and which also displayed a prominent enhancement of presynaptic function that was, likewise, NO-dependent. The most persistent form of LTP studied (evoked by eight trains of theta-burst stimuli) was previously shown to have a strong L-VGCC-dependent component (Raymond and Redman, 2002). Since a delayed presynaptic enhancement is also a feature of LTP_L−VGCC_ (see above), the findings raise the question of whether the NO signaling is linked exclusively to NMDAR activation, or whether it can also participate in LTP_L−VGCC_. The present work attempts to answer this question.

## Materials and Methods

### Animals

Work was compliant with British Home Office regulations on animal use and welfare and was done with the approval of the University College of London ethical review panel. Unless otherwise stated, male, 6–9-week-old, C57/Bl6 mice (Charles River, Kent, UK) were used. Homozygote male, 6–9-week-old, 129sv/C57Bl/6 mice lacking eNOS (Huang et al., 1995) were provided by Dr. Adrian Hobbs (Queen Mary University, London, UK) and age-, sex- and strain-matched wild-type (WT) mice were obtained from Harlan (Wyton, UK) for use as controls. These mice were not derived from the subpopulation of Harlan C57/Bl6 mice that lack α-synuclein (Specht and Schoepfer, 2001), which may be required for normal synaptic transmission (Bendor et al., 2013). The experimenter was blinded to genotype until after the data had been analyzed.

### Special Chemicals

(2R)-2-Amino-5-phosphonopentanoic acid (D-AP5), dimethyl 2,6-dimethyl-4-(2-nitrophenyl)-1,4-dihydropyridine-3,5-dicar-boxylate (nifedipine), (2R)-2-(methylamino)butanedioic acid (NMDA) and (2S)-5-[[amino(nitramido)methylidene]amino]-2-azaniumylpentanoate (L-nitroarginine) were purchased from Tocris Bioscience (Bristol, UK). N-[3-Amino-propyl(propyl)amino]-N-hydroxynitrous amide (PAPA/NO) was obtained from Enzo Life Sciences (Exeter, UK), 2-[(3,4-dimetho-xyphenyl)methyl]-7-[(2*R*,3*R*)-2-hydroxy-6-phenylhexan-3-yl]-5-methyl-1*H*-imidazo[5,1-f][1,2,4]triazin-4-one (BAY 60–7550) from Cayman Chemical (MI, USA) and 1*H*-[1,2,4]oxadiazolo[4,3-a]quinoxalin-1-one (ODQ) from Sigma Aldrich (Dorset, UK). Stock solutions were made in equimolar NaOH (D-AP5, NMDA), equimolar HCl (L-nitroarginine) or DMSO (BAY 60–7550, nifedipine, ODQ) and were diluted at least 1000-fold before use. Nifedipine was prepared freshly on the day of each experiment and applied in the dark. Stock solutions of PAPA/NO were also made freshly using 10 mM NaOH as the solvent, and were stored on ice.

### Hippocampal Slice Preparation

Mice were killed by cervical dislocation and the hippocampi quickly dissected out into ice-cold artificial cerebrospinal fluid (aCSF) containing (in mM): 120 NaCl, 2.5 KCl, 1.3 MgCl_2_, 1 NaH_2_PO_4_, 26 NaHCO_3_, 10 D-glucose and 2 CaCl_2_, equilibrated with 95% O_2_/5% CO_2_ to pH 7.4 (at 30°C). Transverse, 400 μm-thick slices were cut from the middle of the hippocampi using a vibratome (Series 100 Sectioning System, Technical Products International, MO, USA) and then recovered for 1–2 h at room temperature on a nylon net submerged in aCSF that was constantly bubbled with 95% O_2_/5% CO_2_.

### Electrophysiology

A slice was transferred to a submerged recording chamber under a dissecting microscope (Carl Zeiss Ltd., Hertfordshire, UK) and superfused with oxygenated aCSF (1–1.5 ml/min, 30 ± 1°C). The Schaffer collateral/commissural pathway was stimulated using a concentric bipolar electrode and extracellular field EPSPs (fEPSPs) were recorded from the stratum radiatum of CA1 using a borosilicate glass electrode filled with aCSF (final resistance 1–3 MΩ). Synaptic efficacy was quantified using the fEPSP initial slope, which was measured from 20 to 50% of the fEPSP peak amplitude. Baseline stimulation was delivered at 0.033 Hz at an intensity that was set to 40–50% of that necessary to evoke a population spike in the stratum radiatum. Recordings were abandoned and/or data were excluded if the mean fEPSP slope measured during the first and last 5 min of baseline recording differed by >20%. LTP was induced using either a 1-s, 100-Hz tetanus or the protocol described by Cavus and Teyler (1996). In this latter protocol (referred to as 200 Hz burst stimulation), a 200-ms, 200-Hz train of stimuli (i.e., 40 pulses) was delivered 10 times every 5 s at a stimulus intensity that evoked a 0.5–1 mV population spike in the area of the stratum pyramidale adjacent to the recording site in the stratum radiatum and was equivalent to an increase in the baseline stimulus intensity by 29 ± 7% (mean ± SEM; *n* = 15). Field EPSPs were amplified (Axoclamp-2B amplifier, Molecular Devices, CA, USA), low-pass filtered (1 kHz) and sampled using Clampex 10.2 Software (Molecular Devices). Drugs were delivered through the perfusion system. Experiments were interleaved or run simultaneously with controls using two recording chambers.

### cGMP Measurement

The methods used have been reported previously (Pigott et al., 2013). Briefly, hippocampal slices were randomly distributed to flasks of oxygenated aCSF held in a shaking water bath and maintained at 30°C. cGMP accumulation was stimulated using a concentration of NMDA (100 μM) that is maximal for the response in rat hippocampal slices (Hopper and Garthwaite, 2006). An inhibitor of phosphodiesterase-2 (BAY 60–7550, 1 μM) was added 15 min beforehand to increase the cGMP signal-to-noise ratio and when used, the NO synthase inhibitor L-nitroarginine (100 μM) and the NMDA antagonist D-AP5 (20–500 μM) were applied 20 min beforehand. After 2–2.5 min stimulation, the slices were inactivated by submersion in boiling buffer composed of 50 mM tris-HCl and 4 mM EDTA (pH 7.4 at room temperature; 200 μl per slice). cGMP was measured by radioimmunoassay and normalized to the total tissue protein.

### Statistics

Data are mean values ± SEM and were collected using slices from at least three animals. Values of *n* refer to the number of slices used. Statistical analysis used OriginPro 2016 (OriginLab Corporation, MA, USA) or GraphPad Prism 6 Software (CA, USA) and significance was inferred when *p* < 0.05 (statistical power for *t*-tests ranged from 70 to 100% where *p* < 0.05). For LTP, data were normalized to the mean fEPSP initial slope measured over the first 10 min of baseline shown. LTP was induced at the arrow shown in each figure. Values of LTP quoted in the text were measured 55–60 min post-induction and unless otherwise stated, statistical comparisons were made using the mean fEPSP slopes recorded during this time. Inset traces are representative of the mean fEPSP recorded over the times indicated by the numbered bars; stimulus artifacts have been truncated.

## Results

**Figure 1:**
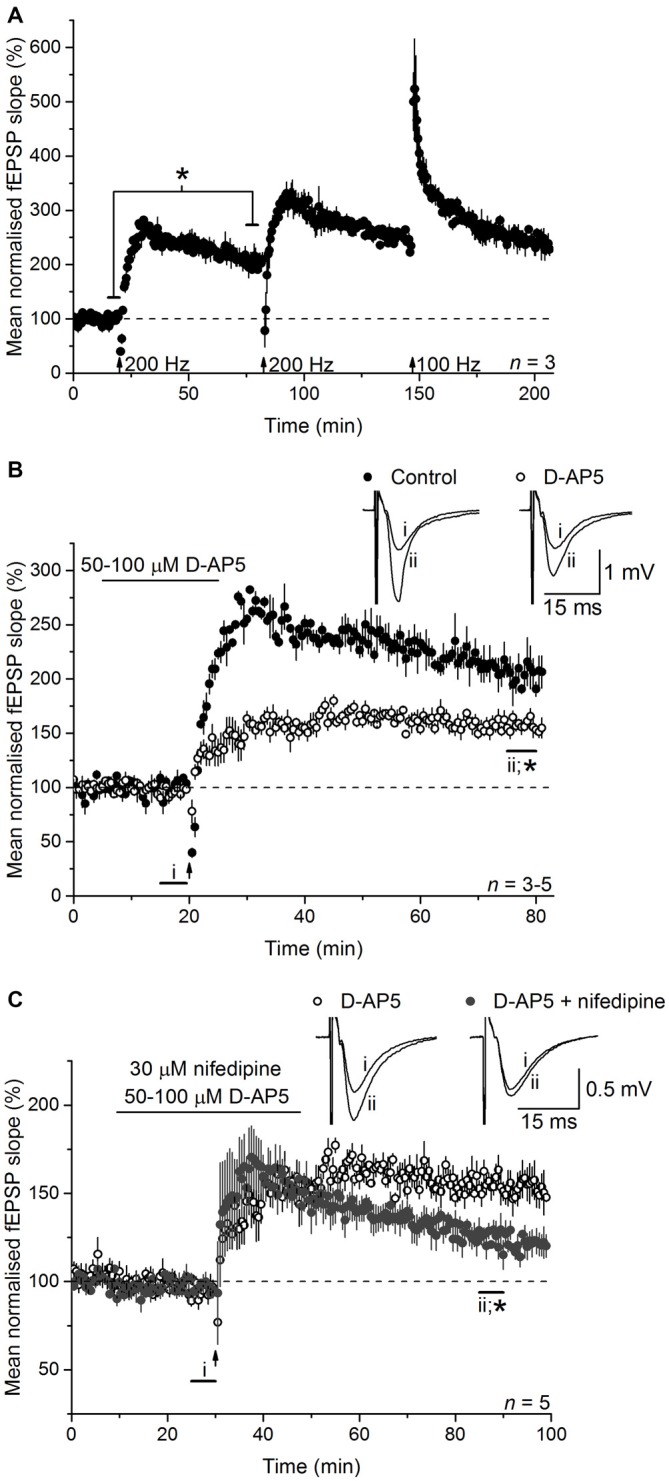
**Long-term potentiation (LTP) induced by 200-Hz burst stimulation has separable NMDA receptor (NMDAR)- and L-type voltage-gated Ca^2+^ channels (L-VGCC)-dependent components. (A)** 200-Hz burst stimulation (delivered at the first arrow) reliably induced a high-magnitude LTP at CA3-CA1 synapses (*paired *t*-test, *p* = 0.019 at 55–60 min post induction compared to the last 5 min of baseline; *n* = 3) that occluded further potentiation by subsequent 200-Hz stimulation (delivered at the second arrow) or a 1-s, 100-Hz tetanus (delivered at the third arrow; repeated measures analysis of variance (ANOVA), *p* = 0.074 at 55–60 min after induction; *n* = 3). **(B)** The NMDA antagonist D-AP5 (50 or 100 μM) significantly reduced the amplitude of the LTP (*p = 0.006 by unpaired *t*-test, 55–60 min post induction), although a stable, significant potentiation persisted (paired *t*-test, *p* = 2 × 10^−4^ compared to the last 5 min of baseline). Note that unfilled circles show the mean of three experiments done with 50 μM D-AP5 (LTP measured 153 ± 5% at 55–60 min after induction) and two with 100 μM D-AP5 (166 ± 8%). **(C)** The L-VGCC inhibitor nifedipine (30 μM) reduced the NMDAR-independent LTP to a dwindling potentiation (*unpaired *t*-test, 55–60 min following induction, *p* = 0.004). All experiments were interleaved. For ease of comparison, the control data shown in **(A)** and the D-AP5-insensitive LTP shown in **(B)** have been re-plotted in **(B,C)**, respectively.

To test the involvement of NO in LTP_L−VGCC_, we initially used an LTP-induction protocol that was reported to generate a mixed potentiation with distinct NMDAR-dependent and L-VGCC-dependent components. This induction protocol (referred to as 200 Hz burst stimulation) reliably generated a high-amplitude LTP at CA3-CA1 synapses in mouse hippocampal slices (Figure 1A). Between 55 and 60 min after induction (an interval used routinely for quantification in the present work), the amplitude amounted to 205 ± 14% of the baseline value. Little or no preceding short-term potentiation was evident, in common with several other findings using a similar burst type of stimulation (Morgan and Teyler, 2001; Bayazitov et al., 2007; Grover et al., 2009; Johnstone and Raymond, 2011). No further enduring potentiation could be generated by repeating the stimulation or by subsequently delivering a standard 1-s, 100-Hz tetanus (when short-term potentiation was evident), suggesting that a single 200-Hz burst stimulation protocol induces an LTP that comprises all available expression mechanisms.

**Figure 2:**
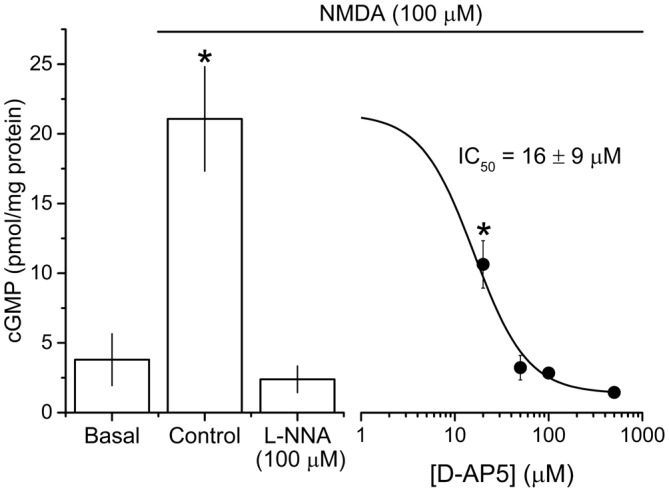
**Potency of the NMDA antagonist D-AP5 against NMDA-evoked cGMP accumulation.** NMDA (100 μM, 2 min) induced a significant increase in cGMP from basal/unstimulated levels that was abolished by pre-incubation with the NOS inhibitor L-nitroarginine (L-NNA, 100 μM, 20 min) and attenuated by the NMDA antagonist D-AP5 (20 min) in a concentration-dependent manner. The IC_50_ value (±SEM) was calculated using the logistic fit shown (adjusted R^2^ of the fit = 0.839). Asterisks (*) mark responses that are significantly different in amplitude from the one obtained in the presence of L-NNA (ANOVA with Dunnett’s *post hoc* test; L-NNA vs. control, adjusted *p* < 1 × 10^−4^; L-NNA vs. 20 μM D-AP5, adjusted *p* = 0.026; *n* = 3–5 slices). The logistic fit in figure was generated using Origin 9.1 (OriginLab Corporation, MA, USA).

To isolate NMDAR-independent LTP, the NMDA antagonist D-AP5 was applied at a concentration (50 μM) that was supramaximal for inhibiting the NMDAR-mediated synaptic potential during 200 Hz stimulation (Grover and Teyler, 1990, 1994). In a further test of particular importance to the present study, we measured the accumulation of cGMP, which is the second messenger for NO, in hippocampal slices exposed to a maximal (100 μM) concentration of NMDA (Hopper and Garthwaite, 2006). The cGMP response was reduced by 50 μM D-AP5 to the same level as was observed using a supramaximal concentration (East and Garthwaite, 1991) of the NO synthase inhibitor L-nitroarginine (Figure 2), implying that NO formation linked to NMDAR activation was effectively abolished by this D-AP5 concentration.

As expected from past reports, following 200 Hz burst stimulation in the presence of D-AP5, a slowly rising potentiation that reached its peak after about 15 min and that was approximately half the control amplitude (158 ± 6%) was observed (Figure 1B). In interleaved experiments, co-application of the L-VGCC inhibitor nifedipine (30 μM) caused a gradual loss of the NMDAR-independent LTP such that, although still on the wane, it had fallen to 125 ± 6% of baseline at the chosen measurement interval (55–60 min post-induction; Figure 1C). The effect of nifedipine observed here is similar in time-course and magnitude to that observed in other studies using either the same compound (Grover and Teyler, 1990; Cavus and Teyler, 1996), or another dihydropyridine-based L-VGCC inhibitor (nitrendipine; Bayazitov et al., 2007), or the phenylalkylamine verapamil (Morgan and Teyler, 1999) or slices from mice lacking the predominant L-VGCC α-subunit in hippocampus, Ca_V_1.2 (Moosmang et al., 2005). Accordingly, the D-AP5-resistant LTP in our experiments qualifies as LTP_L−VGCC_.

**Figure 3:**
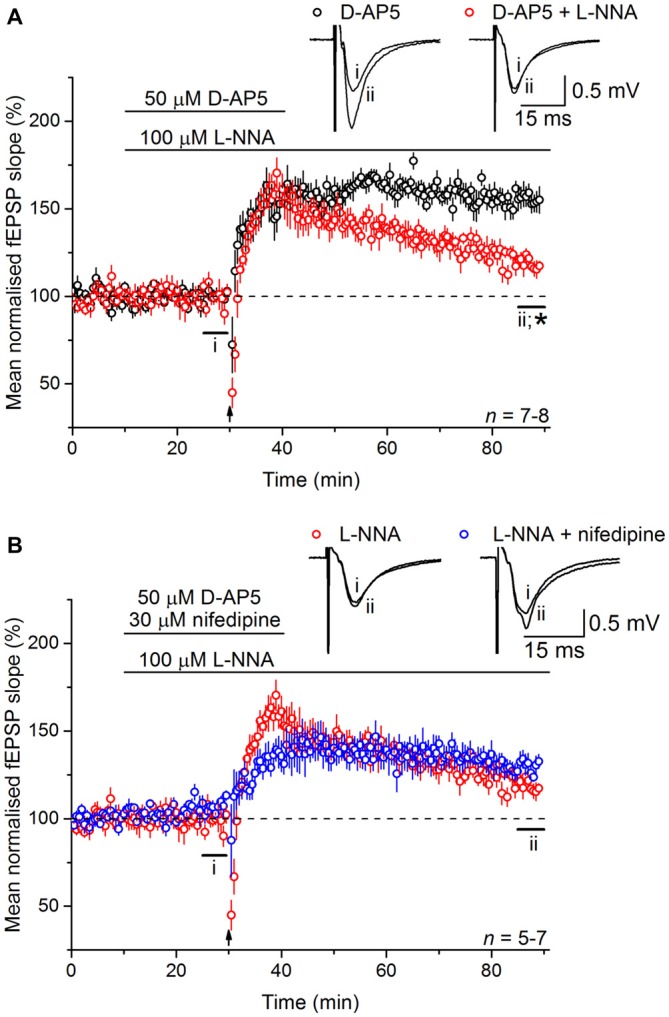
**Nitric oxide (NO) is necessary for LTP_L−VGCC_. (A)** The NMDAR-independent, L-VGCC-dependent LTP (LTP_L−VGCC_; black) was significantly reduced by the NO synthase inhibitor L-nitroarginine (L-NNA, 100 μM; red; *unpaired *t*-test, *p* = 5 × 10^−4^ at 55–60 min post induction). **(B)** No further inhibition could be generated by co-applying the L-VGCC inhibitor nifedipine (30 μM) with L-nitroarginine (unpaired *t*-test, *p* = 0.056 at 55–60 min after 200 Hz stimulation).

To test the involvement of NO in LTP_L−VGCC_, slices were bathed in the NO synthase inhibitor, L-nitroarginine (100 μM). Much like the effect of nifedipine, L-nitroarginine reduced LTP_L−VGCC_ to a dwindling potentiation (119 ± 4%; Figure 3A). Moreover, the effects of nifedipine and L-nitroarginine were mutually exclusive in that the level of LTP in the presence of both inhibitors together (131 ± 5%) was not significantly different from either one alone (analysis of variance (ANOVA), *p* = 0.197; *n* = 5–7; Figure 3B).

**Figure 4:**
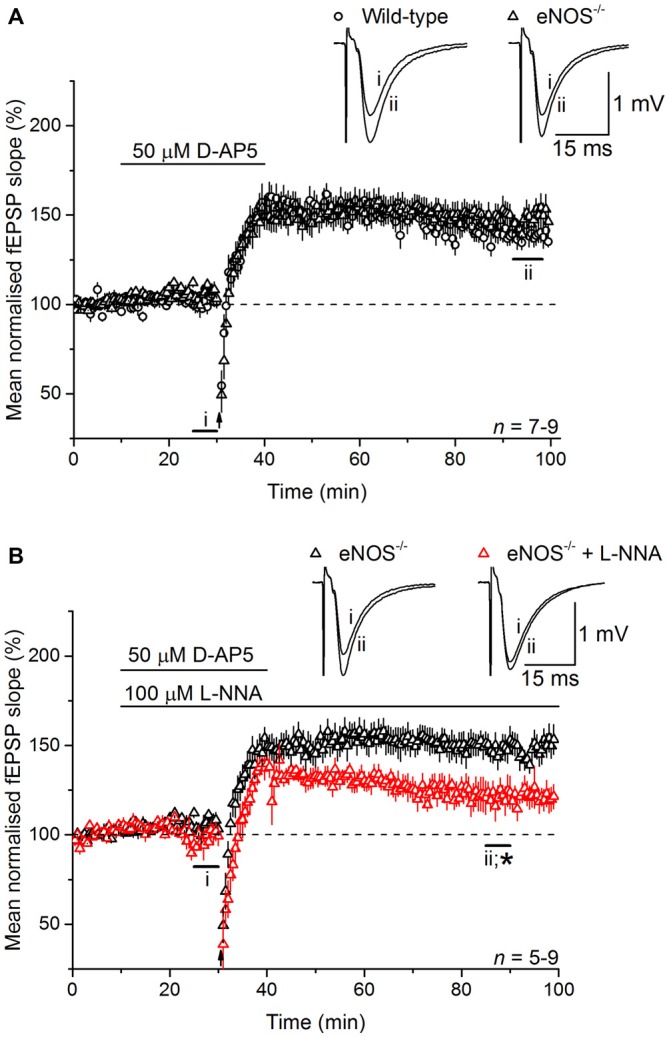
**LTP_L−VGCC_ does not require eNOS. (A)** LTP_L−VGCC_ in slices from eNOS knockout (eNOS^−/−^) mice was indistinguishable from the potentiation observed in slices from wild-type (WT) mice that were matched for age, sex and strain (unpaired *t*-test of mean fEPSP slopes at 55–60 min post induction, *p* = 0.377). **(B)** In slices from eNOS knockout mice, LTP_L−VGCC_ was significantly reduced by the NOS inhibitor L-nitroarginine (L-NNA, 100 μM; *unpaired *t*-test, 55–60 min after induction, *p* = 0.018).

At CA3-CA1 synapses, gene deletion experiments in mice have shown that NO-dependent LTP depends on both endothelial NO synthase (eNOS) and nNOS (Son et al., 1996; Wilson et al., 1997; Hopper and Garthwaite, 2006; Phillips et al., 2008), with the former (in blood vessels) deemed to provide a basal NO tone and the latter a phasic signal in response to the tetanic stimulation itself (Hopper and Garthwaite, 2006). To determine the source(s) of the NO required for LTP_L−VGCC_, and in the absence of NO synthase inhibitors that discriminate usefully between nNOS and eNOS (Pigott et al., 2013), we used mice lacking eNOS. LTP_L−VGCC_ was of normal time-course and magnitude in slices from the knockout mice (Figure 4A) and displayed WT sensitivity to inhibition of NO synthase with L-nitroarginine (122 ± 7%; Figure 4B).

**Figure 5:**
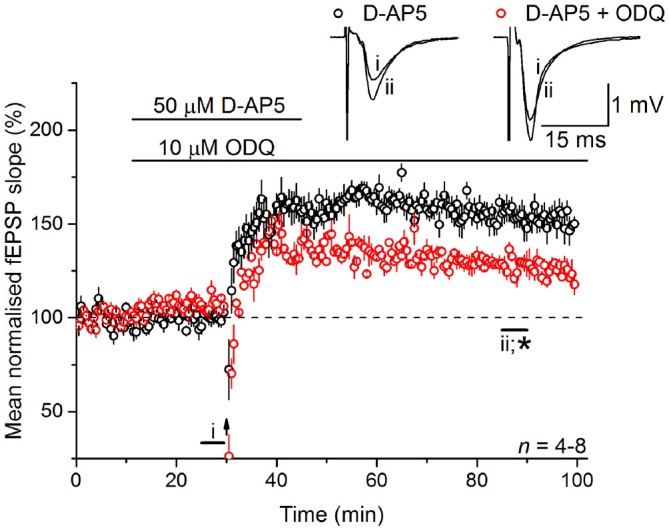
**LTP_L−VGCC_ necessitates activation of the guanylyl cyclase-coupled NO receptor.** LTP_L−VGCC_ was significantly attenuated by an antagonist of the guanylyl cyclase-coupled NO receptor, ODQ (10 μM; *unpaired *t*-test, *p* = 0.008 55–60 min following induction). Experiments were interleaved with those shown in Figure 2A. The control data shown in Figure 2A has been re-plotted here for ease of comparison.

Whilst there are exceptions, physiological NO signal transduction is normally through specialized NO receptors equipped with intrinsic guanylyl cyclase activity, whose activation results in the generation of cGMP (Garthwaite, 2008). Inhibition of guanylyl cyclase-coupled NO receptors with 10 μM ODQ (Garthwaite et al., 1995) gave a similar result to L-VGCC and NO synthase inhibition in that it caused a gradual loss of the LTP (128 ± 4%; Figure 5), indicating that the NO signal in LTP_L−VGCC_ was transduced predominantly via the classical cGMP pathway.

**Figure 6:**
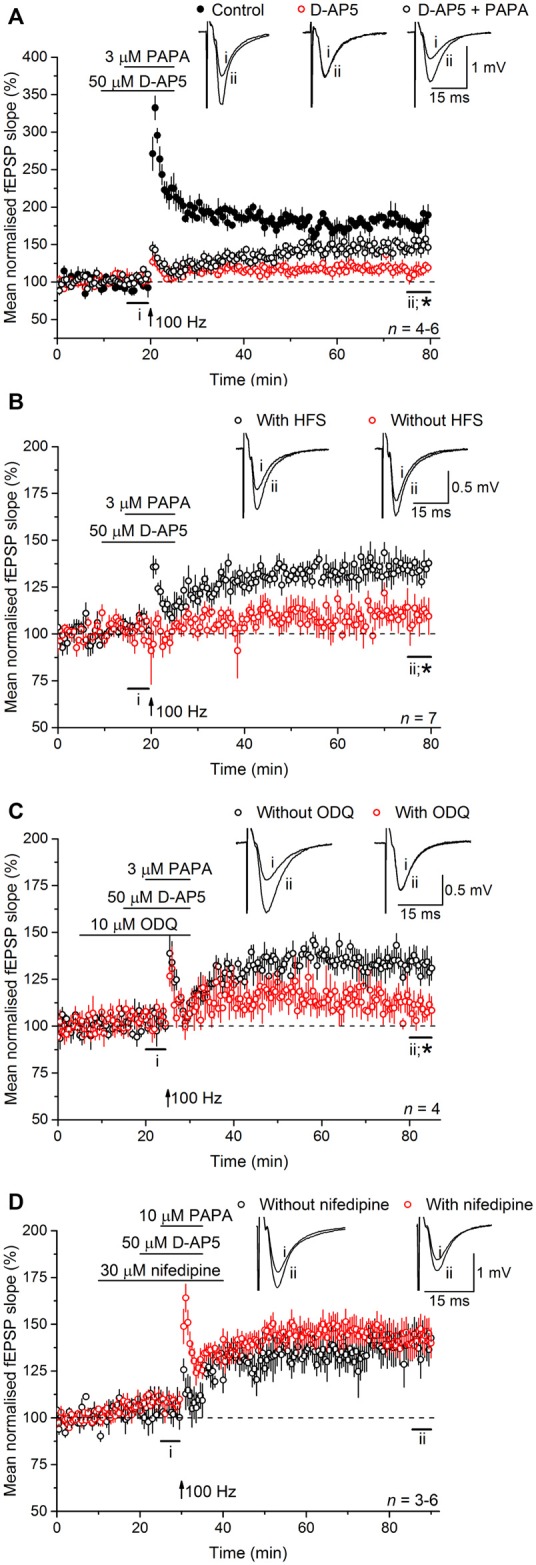
**Exogenous NO, paired with tetanic stimulation, generates a slowly rising potentiation that is resistant to L-VGCC inhibition. (A)** A standard, 1-s, 100-Hz tetanus (delivered at the arrow) generated an LTP (black; paired *t*-test, *p* = 2 × 10^−4^ at 55–60 min after induction compared to the last 5 min of baseline) that was significantly reduced by the NMDA antagonist, D-AP5 (50 μM; red; *unpaired *t*-test, control vs. D-AP5 at 55–60 min after the tetanus, *p* = 2.24 × 10^−5^; paired *t*-test, mean slope in the presence of D-AP5 at 55–60 min after stimulation at 100 Hz vs. the last 5 min of baseline, *p =* 0.292). When paired with the NO donor PAPA/NO (3 μM) in the presence of D-AP5, the tetanus generated a slowly rising, long-lasting potentiation (open circles; paired *t*-test, mean slope in the presence of PAPA/NO at 55–60 min after 100 Hz stimulation vs. the last 5 min of baseline, *p* = 0.008; *unpaired *t*-test, PAPA vs. D-AP5 at 55–60 min after the tetanus, *p* = 0.007). **(B)** The NO donor PAPA/NO (3 μM) had no effect on the mean fEPSP slope under basal conditions (without HFS, red; paired *t*-test, mean slope without HFS at 15–20 min vs. at 75–80 min, *p* = 0.149; *unpaired *t*-test, with HFS vs. without HFS at 55–60 min after the tetanus, *p* = 0.002). **(C)** The potentiation generated by pairing PAPA/NO with the tetanus was significantly attenuated by the guanylyl cyclase-coupled NO receptor antagonist ODQ (10 μM; *unpaired *t*-test, with ODQ vs. without ODQ at 55–60 min following induction, *p* = 0.0184). **(D)** The L-VGCC inhibitor nifedipine (30 μM) had no effect on the amplitude of the NO-induced potentiation (unpaired *t*-test, with nifedipine vs. without nifedipine at 55–60 min post tetanus, *p* = 0.776).

Experiments were then carried out to determine if NO operates upstream or downstream of L-VGCCs. To do so, exogenous NO in the form of the NO donor PAPA/NO was applied in the presence of D-AP5, with or without tetanic stimulation. The concentrations of PAPA/NO used (3–10 μM) elicit a maximal cGMP-dependent axonal depolarization when superfused over rat optic nerve and are orders of magnitude lower than the concentrations yielding toxic amounts of NO (Garthwaite et al., 2006). When PAPA/NO was paired with a standard tetanus (100 Hz for 1 s) in the presence of D-AP5, there developed a slowly rising potentiation that measured 149 ± 10% 55–60 min afterwards (Figure 6A) whereas, under basal stimulation conditions (0.033 Hz), the NO donor was ineffective (115 ± 7%; Figure 6B). The potentiation seen after pairing the tetanus with PAPA/NO was attenuated by inhibiting the guanylyl cyclase-coupled NO receptor with ODQ (10 μM; 109 ± 8%; Figure 6C), whereas it was unaffected by the L-VGCC inhibitor nifedipine (30 μM; 143 ± 8%; Figure 6D) implying that NO was acting via cGMP, but independently of L-VGCCs.

## Discussion

Until now, it had been assumed that the participation of NO in hippocampal CA3-CA1 LTP, which is traditionally regarded as NMDAR-dependent (Collingridge et al., 1983), reflects the functional and molecular coupling between NMDARs and nNOS activity, together with a background input from eNOS in the microcirculation and to our knowledge, the possibility that NO could participate in LTP_L−VGCC_ had not been considered. On the evidence reported here, and under conditions where NMDA-stimulated NO generation was abolished, LTP_L−VGCC_ was found to be entirely dependent on the NO-cGMP pathway in that inhibition of NO synthase or NO-activated guanylyl cyclase had effects on NMDAR-independent LTP that were the same as inhibition of L-VGCCs, and no further loss of LTP took place under combined inhibition of NO synthase and L-VGCCs.

Whilst initially appearing to require types of stimulation of questionable physiological significance (Grover and Teyler, 1990), it soon became clear that a range of burst-type stimuli, and even the traditional single 100 Hz tetanus, could elicit an LTP containing a component that was reliant on L-VGCCs (Cavus and Teyler, 1996; Morgan and Teyler, 2001; Bayazitov et al., 2007; Grover et al., 2009). Moreover, deletion of the principal L-VGCC α-subunit (Ca_V_1.2) in the hippocampus and cortex, which leads to a loss of LTP_L−VGCC_, led to a marked impairment in hippocampus-dependent spatial memory (Moosmang et al., 2005), signifying, along with prior pharmacological evidence (Borroni et al., 2000; Woodside et al., 2004), that LTP_L−VGCC_ has behavioral significance. Impairment in spatial memory is also one of several behavioral abnormalities seen in mice deficient in nNOS (Weitzdoerfer et al., 2004; Tanda et al., 2009; Walton et al., 2013), consistent with LTP_L−VGCC_ and NO being functionally related *in vivo*.

In preceding studies, it was found that the most enduring LTP at CA3-CA1 synapses, induced by a series of 8 theta-burst stimulus trains, was dependent on both L-VGCCs (Raymond and Redman, 2002) and NO (Johnstone and Raymond, 2011), in keeping with a link between the two. That this form of LTP is also partially NMDAR-dependent (Raymond and Redman, 2006), coupled with evidence that a less persistent form of LTP induced by a series of four stimulus trains and having no obvious L-VGCC-mediated component was similarly NO-dependent, led to the suggestion that the NO signal was linked to NMDARs (Johnstone and Raymond, 2011). Our results, do not negate this interpretation, but indicate that there is a previously unconsidered means of generating NO in LTP that may act independently of, or in league with, NMDAR-linked NO formation.

The most probable source of the NO is in neurons because, unlike conventional LTP elicited by a 100 Hz, 1-s tetanus, LTP_L−VGCC_ was unaffected in slices from mice lacking eNOS and because the inducible form of NO synthase, which can be expressed in response to immune challenge, is not detectable in incubated hippocampal slices (Hopper and Garthwaite, 2006). In the hippocampus, nNOS protein is found throughout the neuropil, in pyramidal neurons, and in populations of GABAergic interneurons (reviewed by Hardingham et al., 2013) so, in principle, the NO signal could originate from any of these structures, although the simplest option is that it is derived from the synaptically activated CA1 pyramidal neurons themselves.

An obvious mechanism of NO production would be that the Ca^2+^ influx through postsynaptic L-VGCCs that provides the trigger for LTP_L−VGCC_ (Grover and Teyler, 1990), also stimulates nNOS. An alternative option, that L-VGCCs are downstream of NO, is unlikely given our finding that the potentiation induced by exogenous NO paired with tetanic stimulation under conditions of NMDAR blockade was unaffected by nifedipine. This finding is comparable with earlier results in which exogenous NO or the permeant cGMP analog 8-bromo-cGMP, when paired with a stimulus too weak in itself to induce LTP, evoked a slowly rising NMDAR-independent potentiation in the hippocampus that was resistant to L-VGCC blockade (Zhuo et al., 1993, 1994).

In accordance with L-VGCCs mediating stimulation of nNOS, protein for the principal hippocampal L-VGCC subunit Ca_V_1.2 is concentrated in dendritic spines of CA1 pyramidal neurons (Davare et al., 2001; Obermair et al., 2004; Leitch et al., 2009), where nNOS is also located (Burette et al., 2002). Functionally, L-VGCCs in CA1 dendritic spines can be activated by back-propagating action potentials, although the resulting influx of Ca^2+^ contributes little to the global rise in intraspinal Ca^2+^ (Yasuda et al., 2003; Bloodgood and Sabatini, 2007) so that nNOS would probably need to be located close to the L-VGCC channel mouth to become activated through this route. Of possible relevance here, L-VGCCs, NMDARs and nNOS reportedly lie within 80 nm of each other in lipid rafts in cerebellar granule cell cultures (Marques-da-Silva and Gutierrez-Merino, 2012) and the more persistent, and NO-dependent, forms of CA3-CA1 LTP evoked by multiple trains of theta-burst stimulation require postsynaptic action potential firing during their induction (Phillips et al., 2008; Raymond, 2008). An alternative locus for the interaction would be in the somato-dendritic region, where prominent L-VGCC-mediated increases in intracellular Ca^2+^ are seen following theta-burst stimulation (Raymond and Redman, 2006; Decostanzo et al., 2010) and where, under mild fixation conditions, nNOS immunoreactivity is also found (Burette et al., 2002).

One difficulty with this hypothesis is that there is little evidence for a coupling between L-VGCC and NO release under physiological conditions. In the peripheral nervous system, the generation of NO presynaptically in response to action potentials is primarily regulated by N-type Ca^2+^ channels (Vincent, 2010) and in cerebral cortex slices, the increased NO synthase activity seen on depolarizing neurons by exposure to elevated K^+^ (50 mM) appears to result mainly from Ca^2+^ influx through P-type, rather than L-type, channels (Alagarsamy et al., 1994). On the other hand, L-VGCCs play a major role in NO generation by primary cultures of striatal neurons exposed to NMDA (Rodriguez-Alvarez et al., 1997) or of cerebral cortex exposed to elevated K^+^ (25 mM) or just firing spontaneously (Oka et al., 1999). Hence, although we cannot rule out a more complex mechanism in which NO is synthesized independently of L-VGCCs, a linear sequence of postsynaptic depolarization and opening of L-VGCCs, with the associated Ca^2+^-influx activating nNOS, remains the simplest explanation. It has been speculated that this same sequence underlies an L-VGCC- and NO-dependent form of LTP seen in inhibitory synapses in the thalamus following postsynaptic spike bursting (Sieber et al., 2013).

Given that LTP_L−VGCC_ is induced postsynaptically (Grover and Teyler, 1990) yet is maintained predominantly presynaptically (Zakharenko et al., 2001; Bayazitov et al., 2007), it is tempting to invoke NO as the retrograde messenger implicit in the mechanism. Favoring this role would be the physicochemical properties of NO (Garthwaite, 2016), the differential subcellular locations of nNOS and NO-receptive guanylyl cyclase (Burette et al., 2002), and previous functional evidence (Garthwaite, 2008; Hardingham et al., 2013) but other findings cast doubt over this interpretation. Notably, the more enduring forms of LTP induced by multiple theta-burst trains, which displayed NO-dependent and protein synthesis-dependent presynaptic changes, were inhibited by postsynaptic application of an impermeant protein synthesis inhibitor, as were the associated alterations in presynaptic function (Johnstone and Raymond, 2013). Although L-VGCCs contribute markedly only to the longer lasting variety of these forms of LTP generated by 8 theta-burst trains (Raymond and Redman, 2002), the result suggests that NO might be acting postsynaptically in LTP_L−VGCC_ and that the retrograde messenger is another molecule, such as BDNF (Leal et al., 2015) which shares an overlapping role with NO in hippocampal CA1-CA3 LTP elicited by theta-burst stimulation (Lessmann et al., 2011).

Of relevance to a possible postsynaptic site of NO action in LTP_L−VGCC_ is the knowledge that L-VGCCs are important in linking neuronal activity to changes in gene expression, a process that is crucial for long-lasting CA3-CA1 LTP and one that relies on a sustained phosphorylation of the transcription factor cAMP response element binding protein (CREB), probably through a pathway involving extracellular signal-regulated kinase (ERK; Murphy et al., 1991; Impey et al., 1996; Kanterewicz et al., 2000; Wu et al., 2001; Moosmang et al., 2005). Abundant evidence indicates that the NO-cGMP pathway regulates ERK activity, CREB phosphorylation, and subsequent gene expression in many different cell types, although the detailed mechanisms are yet to be clarified (Pilz and Broderick, 2005). In the hippocampus, cGMP accumulation has been observed immunohistochemically in pyramidal cell bodies and primary dendrites in response to exogenous NO (Bartus et al., 2013) and patterns of stimuli producing late-phase, NO-dependent LTP also evoked phosphorylation of CREB in postsynaptic CA1 neurons through a mechanism dependent on cGMP-dependent protein kinase (PKG; Lu et al., 1999). In the same study, pairing a subthreshold tetanus with 8-bromo-cGMP resulted in an enduring CREB phosphorylation that was partially blocked if activation of ERK was inhibited. Moreover, the potentiation produced by pairing a weak tetanus with 8-bromo-cGMP was markedly reduced by inhibiting PKG, protein synthesis, or transcription (Lu et al., 1999). Mice lacking Type 1 PKG also show a defect in late-phase, protein synthesis-dependent LTP (Kleppisch et al., 2003). Given the convergence between the NO-cGMP-PKG-gene expression pathway and the L-VGCC-gene expression pathway, an interplay between the two in postsynaptic CA1 neurons, leading to the synthesis of new proteins needed for later-phase LTP may explain the role of the NO-cGMP pathway in LTP_L−VGCC_.

## Author Contributions

All authors listed have made a substantial, direct, and intellectual contribution to the work, and approved it for publication.

## Funding

This work was funded by The Wellcome Trust (UK; grant number 081512/Z/06/Z) and a studentship from the Biotechnology and Biological Sciences Research Council (UK).

## Conflict of Interest Statement

The authors declare that the research was conducted in the absence of any commercial or financial relationships that could be construed as a potential conflict of interest.
